# High uptake of Intermittent Preventive Treatment of malaria in pregnancy is associated with improved birth weight among pregnant women in Ghana

**DOI:** 10.1038/s41598-019-55046-5

**Published:** 2019-12-13

**Authors:** Isabella Quakyi, Bernard Tornyigah, Pascal Houze, Kwadwo A. Kusi, Nathaniel Coleman, Guillaume Escriou, Amos Laar, Michel Cot, Julius Fobil, Gloria Quansah Asare, Philippe Deloron, Abraham K. Anang, Gilles Cottrell, Michael F. Ofori, Nicaise Tuikue Ndam

**Affiliations:** 1 Université de Paris, MERIT, IRD, F-75006 Paris, France; 2 Department of Biological Environmental and Occupational Health Sciences, School of Public Health, College of Health Sciences, University of Ghana, Legon, Ghana; 3 Department of Immunology, Noguchi Memorial Institute for Medical Research, College of Health Sciences, Legon, Ghana; 4 Service de biochimie générale, Hôpital universitaire Necker-Enfants Malades, AP-HP, 149 rue de Sèvres, 75015 Paris, France; 5 Unité de Technologies Biologiques et Chimiques pour la Santé (UTCBS), Paris 5-CNRS UMR8258 Inserm U1022, Faculté de Pharmacie, Université Paris Descartes, Paris, France; 6 Department of Parasitology, Noguchi Memorial Institute for Medical Research, College of Health Sciences, University of Ghana, Legon, Ghana

**Keywords:** Malaria, Epidemiology

## Abstract

Despite the clinically proven advantages of intermittent preventive treatment of malaria in pregnancy (IPTp) with sulfadoxine-pyrimethamine (SP), utilisation has been low in many African countries. To increase uptake and achieve the desired effect, the World Health Organization revised the policy to a monthly administration. Assessing the coverage and impact of the revised policy on pregnancy and neonatal outcomes is, therefore, a necessity. A 2-parallel cross-sectional hospital-based study was carried out among pregnant women attending first antenatal care (ANC) and delivery. Maternal and cord blood samples were assayed for malaria parasites by quantitative PCR targeting both the 18S rDNA and the acidic terminal segment of *Plasmodium falciparum* var genes, and plasma SP levels were measured by liquid chromatography coupled to tandem mass spectrometry. Parasite prevalence was similar between the two study sites but decreased significantly between the first ANC (9% or 43%) and delivery (4% or 11%) based on the qPCR target. At delivery, 64.5% of women received ≥3 IPTp-SP dose, 15.5% received 2 doses and 6% had 1 dose. Taking ≥3 IPTp-SP doses was associated with an average birth weight increase of more than 0.165 kg. IPTp-SP uptake was associated with plasma SP level at delivery (OR = 32.3, p ≤ 0.005, 95% CI (13.3;78.4) for those that reported ≥3 IPTp-SP doses) while the same trend of improved birth weight was observed with high plasma SP levels. The new IPTp policy is well implemented and well utilised by women in the sites considered in this study and translates to the improved birth weight observed. This study confirms the interest and the clinical benefit expected from this policy change.

## Introduction

Pregnant women continue to be at increased risk of *Plasmodium falciparum* infection, and thus its harmful effect on both mother and foetus^1^. In sub-Saharan Africa, where the disease is still highly endemic, primigravidae and HIV infected persons represent two of the groups most at risk of infection and the harmful consequences associated with it^2,3^. The World Health Organization (WHO) recommends use of insecticide-treated nets, effective case management, and intermittent preventive treatment of malaria in pregnancy (IPTp) to control and prevent pregnancy-associated malaria (PAM) in sub-Sahara Africa. IPTp was initially a 2-dose regimen of sulfadoxine-pyrimethamine (SP) to HIV-naïve pregnant women^4,5^. Despite the several established advantages of IPTp-SP, even across regions with a wide range of SP-resistant parasites^6–11^, uptake of the service by pregnant women was still low compared to ANC visits^6,12,13^. This persistently low coverage in part resulted in a call to action by WHO and its partners, intended to increase coverage to meet the 2010 target of 80% IPTp-SP coverage^14,15^. In addition to this call, other studies revealed a three-course or monthly IPTp-SP administration to be more effective in reducing low birth weight than the original two-course policy^16,17^. In 2012, the WHO revised the policy to a monthly administration of IPTp^18^. The main goal of this policy change, which was to increase the doses of IPTp during pregnancy, is also dependent on how early women appear at first antenatal care (ANC) visit and the rate of use of these prenatal consultations. Like most malaria-endemic countries in Africa, Ghana adopted and implemented this revised policy, and this has presented an opportunity for a preliminary assessment of the coverage achieved by the new policy and its impact on maternal and neonatal birth outcomes.

## Results

### Study population characteristics

Overall, 1922 pregnant women aged between 18 and 45 years were recruited in the district hospitals of Maamobi and Kpone on-Sea respectively. This comprises of 987 women at first ANC and 935 women at delivery (Table 1). Among the women recruited at both first ANC and at delivery, participants’ characteristics were similar between the two study sites, except for data on G6PD deficiency at delivery, which was 12.9% (82/635) at Maamobi but the corresponding data were not available at Kpone on-Sea.

**Table 1:** Study population characteristics.

Characteristics	1st ANC visit		Delivery	
	Maamobi (n = 490)	Kpone on-Sea (n = 497)	Maamobi (n = 635)	Kpone on-Sea (n = 300)
Age^#^	27.28 ± 5.98	27.27 ± 5.83	28.13 ± 5.57	26.67 ± 5.32
**Marital Status**^**†**^				
Single	123 (25.2%)	37 (7.4%)	141 (22.2%)	44 (14.7%)
Co-habiting	46 (9.4%)	141 (28.4%)	22 (3.5%)	67 (22.3%)
Married	320 (65.4%)	319 (64.2%)	460 (72.4%)	189 (63%)
**Educational level**^**†**^				
None	48 (9.9%)	44 (8.9%)	68 (10.7%)	31 (10.3%)
Basic education	285 (59.0%)	337 (67.8%)	249 (39.2%)	197 (65.7%)
Middle education	103 (21.3%)	87 (17.5%)	147 (23.2%)	53 (17.7%)
Higer education	47 (9.7%)	29 (5.8%)	62 (9.8%)	19 (6.3%)
**Employment Status**^**†**^				
Yes	389 (79.4%)	375 (75.5%)	496 (78.1%)	232 (77.3%)
No	101 (20.6%)	122 (24.5%)	138 (21.9%)	68 (22.7%)
**Gravidy/Parity**^**†**^				
Primigravidae	126 (25.7%)	123 (24.7%)	121 (19.1%)	75 (25%)
***Pf*. infection diagnosed**				
By 18SqPCR	16 (12.7%)	13 (10.6%)	5 (4.1%)	2 (2.7%)
By ATSqPCR	50 (39.7%)	58 (47.1%)	35 (28.9%)	10 (13.3%)
Multigravidae	364 (74.3%)	374 (75.3%)	513 (80.9%)	225 (75%)
***Pf*. infection diagnosed**				
By 18SqPCR	30 (8.2%)	31 (8.3%)	23 (4.5%)	7 (3.1%)
By ATSqPCR	159 (43.7%)	156 (41.7%)	45 (8.8%)	16 (7.1%)
Gestational age at first ANC^#^	18.4 ± 6.89	17.9 ± 7.13	18.9 ± 7.29	18.3 ± 6.95
**Bednet possession**^**†**^				
Yes	293 (59.8%)	214(43.1%)	498 (78.4%)	272 (90.7%)
No	197 (40.2%)	283 (56.9%)	137 (21.6%)	28 (9.3%)
**Bednet usage**^**†**^				
Always	93 (31.7%)	141 (65.9%)	187 (37.5%)	141 (51.8%)
sometimes	132 (45.1%)	55 (25.7%)	206 (41.4%)	93 (34.2%)
Seldom	68 (23.2%)	18 (8.4%)	105 (21.1%)	38 (14.0%)
HB^x^	10.76 ± 5.98	11.06 ± 5.83	10.86 ± 1.86	11.10 ± 1.12
**Anaemia status**^**†**^				
Severe anaemic (Hb <8.0 g/dL)	25 (5.1%)	17 (3.4%)	44 (7.1%)	1 (0.4%)
Anaemic (11.0< Hb ≥8.0 g/dL)	229 (46.7%)	198 (39.8%)	263 (42.4%)	126 (44.5%)
Peripheral *Pf* detection by microscopy^†^	17 (3.5%)	19 (3.8%)	15 (2.4%)	ND
Placenta *Pf* detection by microscopy^†^			2 (0.3%)	0 (0%)
**G6PD status**^**†**^				
Defect			82 (12.9%)	ND
Normal			553 (87.1%)	
**Birth Outcome**^**†**^				
Low birth weight <2500 g			29 (4.6%)	12 (4.0%)

^#^Mean ± SD; ^†^n (%); *p-value < 0.005; G6PD defect comprises partial and full deficit, ND not done.

### *P. falciparum* prevalence in the ANC cohorts

*P. falciparum* parasite prevalence detected by microscopy at first ANC was 3.5% and 3.8% at Maamobi and Kpone on-Sea, respectively. When assessed by 18Sq qPCR, parasite prevalence was 9.4% (46/490) at Maamobi and 8.9% (44/497) at Kpone on-Sea. Parasite prevalence rate increased at both sites when assessed by ultrasensitive ATSqPCR assay; 42.2% (207/490) at Maamobi and 43.1% (214/497) at Kpone on-Sea (Table 2). Thus before first IPTp-SP administration at ANC, 5.9% and 5.0% of pregnant women were submicroscopically infected at Maamobi and Kpone on-Sea respectively.

**Table 2:** *P.falciparum* prevalence among different sample types by both 18SqPCR and ATSqPCR.

Characteristics	1st ANC		Delivery	
	Maamobi (n = 490)	Kpone on-Sea (n = 497)	Maamobi (n = 634)	Kpone on-Sea (n = 300)
Peripheral infection*				
By 18SqPCR	46(9.4%)	44(8.9%)	28(4.4%)	9(3.0%)
By ATSqPCR	207(42.2%)	214(43.1%)	80(12.6%)	26(8.7%)
**Parasite density**^**#**^				
By 18SqPCR	21460 ± 42907	27484 ± 74382	27847 ± 130059	21943 ± 43029
**Placental infection**				
By 18SqPCR			16(2.5%)	5(1.7%)
by ATSqPCR			44(6.9%)	11(3.7%)

*n (%); ^#^Mean ± SD;

### *P. falciparum* prevalence in the delivery cohorts

At delivery, parasite prevalence by microscopy was 2.4% (15/635) and 0% at Maamobi and Kpone on-Sea, respectively. Prevalence of infections increased to 4.4% (28/635) at Maamobi and 3% (9/300) at Kpone on-Sea by 18SqPCR, and remained high for ATSqPCR; 12.6% (80/635) at Maamobi and 8.7% (26/300) at Kpone on-Sea. However, placental infection was observed in 0.3% (2/635) of women at Maamobi and 0% of women at Kpone on-Sea by microscopy. Placental infection prevalence rates by 18SqPCR were 2.5% (16/635) at Maamobi and 1.7% (5/300) at Kpone on-Sea, while infection prevalence when tested by ATSqPCR was 6.9% (44/635) at Maamobi and 3.7% (11/300) at Kpone on-Sea (Table 2). When we compared data from the 18SqPCR and the ATSqPCR, there was 84.6% concordance between the two methods used as all infections detected by the 18SqPCR was also detected by the ATSqPCR except one. However, there was a 15.3% discordance between the two methods mainly due to detection of infection by ATSqPCR among the 18SqPCR negatives (Table 3).

**Table 3:** The concordance and discordance between infections detected targeting the 18 SqPCR and the ATS qPCR.

18SqPCR	ATS qPCR		Row total
	Positive	Negative	
Positive	134	1	135
Negative	432	2253	2685
Column total	566	2254	2820

### Coverage and impact of IPTp in the delivery cohorts

**Figure 1:**
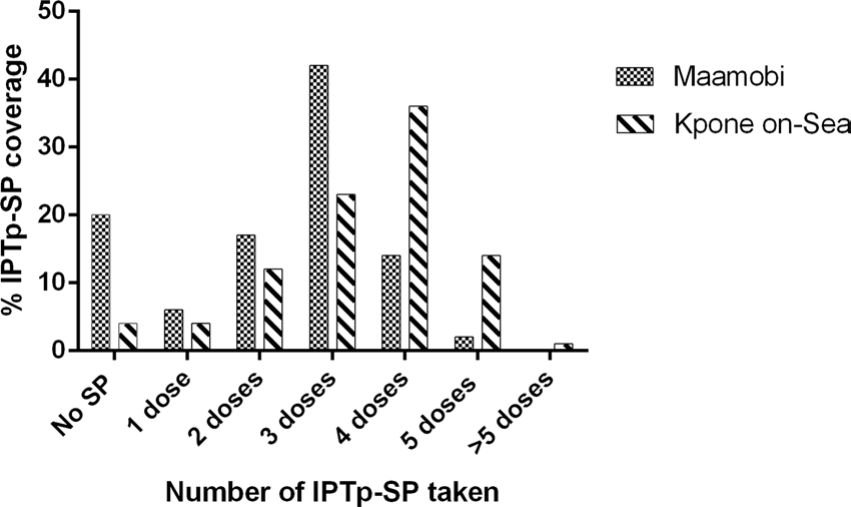
IPTP-SP coverage among delivery women in Maamobi (grey bars) and Kpone on-Sea (stripped bars).

At delivery, information on IPTp-SP uptake was taken from the participants ANC booklets as a reliable source because of the practice of directly observed treatment. Among the 935 women recruited at delivery, 603 (64.5%) had taken ≥3 IPTp-SP doses, 143 (15.3%) had received 2 IPTp-SP doses while 53 (5.7%) had taken 1 IPTp-SP dose. One hundred and thirty-six (14.5%) did not receive any IPTp-SP. When the data were stratified by site, of the 635 participants at Maamobi, 365 (57.5%) had taken ≥3 doses, 106 (16.7%) had taken 2 IPTp-SP doses and 39 (6.1%) had taken 1 IPTp-SP dose. One hundred and twenty-five (125) participants did not receive any IPTp-SP, and among them 82 (65.6%) were disqualified at the hospital due to glucose-6-phosphate dehydrogenase (G6PD) deficiency detected by a nitroblue tetrazolium test (Fig. 1).

At Kpone on-Sea, 238 of the 300 participants (79.3%) received ≥3 IPTp-SP doses, 37 (12.3%) received 2 IPTp-SP doses while 14 (4.7%) received 1 IPTp-SP dose. Eleven (11) participants reported no IPTp-SP uptake due to non-attendance to ANC (Fig. 1).

Women who took ≥3 IPTp-SP doses had a lower risk of *P. falciparum* infection detected by qPCR, compared to those who received only 1 or 2 doses, (OR = 0.72, p = 0.36 at Maamobi; OR = 0.69, p = 0.59 at Kpone on-Sea,) (Table 4). In contrast, the overall linear regression analysis showed that uptake of ≥3 IPTp-SP doses increase the average birth weight by more than 0.165 Kg. This increase was only statistically significant at Maamobi (P < 0.005) although the same trend was visible at Kpone on-Sea (Table 5). This effect was lower and not significant in women taking only 1 or 2 IPTp-SP doses although an increase in birth weight of about 0.72 Kg was observed as compared with women who did not receive any IPTp-SP treatment.

**Table 4:** Relationship between *P. falciparum* infections detected by ATSqRT-PCR and IPTp-SP^a^ uptake using a multivariate logistic regression.

Risk factor	Odds Ratio	95% confidence interval	P value
**Overall P.f**.^*^ **infection diagnosed by qPCR at delivery (overall)**			
<3 IPTp-SP uptake	Reference		
≥3 IPTp-SP uptake	0.71	(0.38; 1.32)	0.29
multigravidae	Reference		
Primigravidae	0.81	(0.37; 1.78)	0.6
**P.f. infection diagnosed by qPCR at Maamobi**			
≥3 IPTp-SP uptake	0.72	(0.36; 1.45)	0.36
Primigravidae	0.71	(0.27; 1.87)	0.49
**P.f. infection diagnosed by qPCR at Kpone on- Sea**			
≥3 IPTp-SP uptake	0.69	(0.18; 2.70)	0.59
Primigravidae	1.09	(0.28; 4.26)	0.9

^a^comparison of ≥3 IPTp-SP versus <3 doses taken; ^*^Plasmodium falciparum.

**Table 5:** Relationship between SP uptake and birth weight by multivariate linear regression.

	Coefficients (Kg)	95% confidence interval	p-value
No SP uptake	Reference		
**Kpone on-Sea**			
1 or 2 IPTp-SP uptake^a^	0.072	(−0.22; 0.37)	0.63
≥3 IPTp-SP uptake^b^	0.201	(−0.07; 0.48)	0.15
Primigravidae	−0.107	(−0.23; 0.01)	0.08
**Maamobi**			
1 or 2 IPTp-SP uptake	0.075	(−0.03; 0.18)	0.18
≥3 IPTp-SP uptake	0.166	(0.07; 0.26)	<0.005
Primigravidae	−0.188	(−0.28; −0.10)	<0.005

^a^SP uptake of less than 3 doses (1 or 2 SP uptake) reflecting the old IPTp policy recommendation; ^b^SP uptake of 3 or more doses reflecting the 2012 recommendation.

### Sulfadoxine-pyrimethamine concentration measurement

To assess and confirm uptake of SP by pregnant women, we measured SP residual levels among a sub-group of participants from both the ANC and delivery cohorts. For ANC, this was to assess whether pregnant women attending their first ANC at the study sites had taken any SP. In the first ANC group, we measured SP residual levels among 353 and 408 plasma samples from Maamobi and Kpone on-Sea, respectively. Of the samples analysed at Maamobi, sulfadoxine was detected in 99 (28%) of them, with median concentration of 3777 mg/ml and interquartile range (IQR: 263–38335), while we detected pyrimethamine in 54 women (15.3%) and median concentration of 224 ng/ml (IQR: 18.75–410.3). At Kpone on-Sea, sulfadoxine and pyrimethamine were detected in 10 (2.5%) plasma samples at median concentration of 2160 mg/ml (IQR: 256.8–4378) and in 1 (0.2%) plasma sample at a concentration of 216 ng/ml, respectively. Women in whom we detected pyrimethamine were also sulfadoxine-positive, however sulfadoxine was detected in more women. So, we considered the presence of sulfadoxine as a proxy for SP in the analyses.

In the delivery group, we measured SP residual levels in peripheral and also in cord blood to explore whether the amount of SP observed in the mother’s peripheral blood could be detected in the cord compartment. Assays were performed in 406 peripheral and 365 cord plasma samples from Maamobi compared to 130 paired peripheral and cord plasma samples from Kpone on-Sea. At Maamobi, we detected sulfadoxine among 306 (75.4%) of peripheral plasma samples at median concentration of 3296 mg/ml (IQR: 631.8–9748) compared to 265 (72.6%) cord plasma samples at median concentration of 2626 mg/ml (IQR: 625.5–8403). Pyrimethamine was detected in peripheral plasma of 117 (28.8%) women at median concentration of 18 ng/ml (IQR: 12–32), and 49 (13.4%) cord plasma samples at concentration of 25 ng/ml (IQR: 13–25). At Kpone on-Sea, we detected sulfadoxine residual levels among 114 (87.7%) peripheral plasma at median concentration of 10945 mg/ml (IQR: 3169–27328) and 113 (87.7%) cord samples at median concentration of 10841 mg/ml (IQR: 4062–27798). Besides, we detected pyrimethamine residual levels among 77 (59.2%) peripheral plasma at median concentration of 27 ng/ml (IQR: 13.3–80.5) and 55 (42.3%) cord samples at median concentration of 50 ng/ml (IQR: 20–120).

SP level in both maternal peripheral and cord plasma was significantly associated with IPTp-SP uptake (Table 6). When the plasma SP levels in the maternal peripheral blood at delivery were categorized into quartiles, the same pattern of improvement in birth weight was observed for women with high concentrations of SP in their blood at delivery. When the data were stratified based on site, this significant association was only observed at Maamobi (Data not shown).

**Table 6:** Relationship between IPTp-SP uptake and residual SP^a^ level measured in the blood at delivery.

SP measurement	OR^b^	95% confidence interval	P value
Primigravidae	0.67	(0.32; 1.41)	0.29
**IPTp uptake**			
1 or 2 IPTp-SP doses^*^	18.9	(6.5; 54.6)	<0.0001
≥3 IPTp-sSP doses taken^#^	32.3	(13.3; 78.4)	<0.0001
Site	0.9	(0.4; 1.8)	0.8

^a^sulfadoxine as a proxy for SP categorised into binary variables (either high or low SP level) base on the median; ^b^Odds-Ratio, *Participants that reported to have received 1 or 2 SP doses, #Participants that reported to have received ≥3 SP doses. The reference classes are the multigravidae, the women who reported no dose of SP taken, and Maamobi site.

## Discussion

Despite the recommendation and implementation of IPTp-SP in some countries in tropical Africa some decades ago, pregnant women are still affected by malaria disease and its effect on their neonates. This is partly due to the low utilisation of the IPTp policy irrespective of high coverage of ANC visits^5,19^. To address this, WHO revised the policy to a monthly administration of SP to reduce inadvertent low or non-uptake of SP^18^. Thus, determining the coverage and effectiveness of this new policy is of utmost importance to its sustainability or otherwise across the different SP-resistant areas in tropical Africa.

In this study, we enrolled two different sets of pregnant women; those who were visiting the health centres for their first antenatal consultation and those coming to deliver at the health centres. The design of this study makes it possible to better capture the characteristics of women appearing at their first ANC and also to determine the use of the services set up for malaria prevention in the delivery cohort. The study design also makes it possible to limit the introduction of potential biases on the envisaged observations. The first observation is the early arrival of women at first ANC, at 18 weeks of pregnancy on average at both study sites, the earliest of which is at 11 weeks. This observation is important and very reassuring for the policy change. Our data which shows over 60% of pregnant women taking ≥3 IPTp-SP doses, together with other recent studies in Ghana that report between 87 and 99% IPTp-SP uptake of ≥3 IPTp-SP doses^20,21^ indicate a high IPTp coverage across Ghana. The observed satisfactory coverage indeed results from the effective campaigns by the Ghana Health Service (GHS) for the IPTp-SP policy. Measuring SP molecules in the blood of women under IPTp corroborated the documented information on treatment received by the participants.

A high proportion of women (more than 75%) with detectable SP in both peripheral and cord plasma at delivery is also indicative of a high IPTp-SP coverage in Ghana as expected by the new policy. Further, this clearly shows that SP can cross the placenta barrier and raises the question whether these high doses of SP that correlated with that of the mother’s compartment are appropriate for the condition of the fetus. However, future studies on the impact of prolonged use of SP in IPTp on the health of the fetus are needed. This is very interesting as this biological measurement correlated with the number of SP doses recorded when the measurement was adjusted for ANC attendance. However, we detected SP in an unexpected and relatively high proportion of women (28%) visiting the urban site for their first ANC, which falls out of line with their declaration. As we did not find this in the peri-urban site; we cannot exclude the hypothesis of possible unreliable disclosure due to self-medication or a first undeclared visit in a private clinic. Our possibilities of verifying what these women in this group disclosed before their first ANC attendance at the study health centres are minimal. Thus, the use of this analytical tool could lead to definition of a follow-up marker for SP uptake.

The significant finding of this study strongly confirms increased birth weight associated with increased SP uptake as recommended by the revised policy. In this case uptake of ≥3 IPTp-SP doses improves birth weight by more than 0.165 Kg. *P. falciparum* infection (either microscopically or sub-microscopically) during pregnancy is one of the major factors underlying low birth weight, and several studies have shown the beneficial effect of IPTp-SP on the birth weight with some indication of a dose-dependent relationship with birth weight^13,16,22^. The low prevalence rates of infections observed at delivery compared with those amongst the first ANC group is an indication of IPTp-SPs’ curative and prophylactic effect as a whole. However, the fact that taking ≥3 doses of IPTp-SP did not show a particular impact on the infection rate at delivery probably suggests that this variable does not provide information on all infections that would have been prevented since initiation of IPTp. This variable can only measure infections observed at delivery which may include more recent infections, while the indirect effect of IPTp that was visible on the improvement of birth weight would better reflect a broader window of coverage.

Sulfadoxine like other sulfonamides is a broad-spectrum antibiotic that is known to likely exert an inhibitory effect against potential non-malarial infections such as sexually transmitted and reproductive tract infections that can cause low birth weight and preterm birth. Thus, SP uptake can sufficiently curb these infections and indirectly improve birth weight as observed elsewhere^22^. This could partially explain why in areas where SP efficacy has reduced, SP continued to improve birth weight outcomes. The effect size is similar in both sites but probably due to lack of power, statistical significance was only achieved at the Maamobi site. A possible explanation may lie in the power of analysis facilitated by a larger sample size of participants that reported of not receiving any IPTp-SP at Maamobi due to G6PD status compared to Kpone on-Sea where virtually every participant received SP, or other possible factors such as maternal nutrition, malaria history or other infections that may be specific to the site but which we are unaware of.

In our study populations the prevalence of microscopically detectable *Plasmodium* infections was low (less than 4%), however, the use of qPCR revealed the huge difference in the proportion of women who were parasite-positive with microscopy and PCR. The high sensitivity of the 18 S qPCR (detection limits of 0.5–3 parasites/µl of blood) and ATS-qPCR (0.06–0.15 parasites/µl of blood)^23^ has been an asset for optimal detection of these infections that are missed by conventional microscopy with a detection limit of only 50 parasites/µl of blood^24^. The reasons for low placental infections compared to peripheral infections are not known, it is possible that all the peripheral infections detected are not necessarily of placental type. Although densities of infection in the placenta are generally higher compared to the peripheral blood, the asymptomatic carriage of certain non-placental type peripheral infections cannot be ruled out. The use of a more sensitive detection PCR in this study, such as ATSqPCR which not only numerically recognizes more targets on the parasite genome than do the 18SqPCR, but also the fact that it was expended over 45 cycles of amplification, allowed to detect these populations probably more prevalent at lower densities.The study demonstrates that low-density parasitemia is very common in high malaria-endemic areas.

To conclude, the new IPTp policy is well implemented and utilised by pregnant women in our study sites confirmed by plasma SP level measurements. Our findings also showed that ≥3 SP administrations of IPTp were associated with increased birth weight as compared to the previous standard 2-dose regimen. Also, as in any observational field study, we did not control for all possible confounding factors. At the same time, this study also reveals that despite the reduction of adverse effects on birth weight by administering of more doses of IPTp-SP, malaria infections remain a threat outside the window of application of IPTp, mainly before women present to antenatal care. Therefore, the need to complement this strategy with an approach that can protect women from infections in the first trimester of pregnancy, such as the use of a vaccine, remains a necessity.

## Methods

### Study population and design

The study was a cross-sectional hospital-based survey conducted in two distinct communities in southern Ghana; Kpone on-Sea, a semi-urban community and Maamobi, an urban community about 50 Km apart. Malaria transmission in both communities is perennial, with two peaks in April–July and September–November. However, the entomological inoculation rate (EIR) at Kpone on-Sea is 62.1^25^ while that of Maamobi is 44.7^26^. At Kpone on-Sea, primary health care is mainly provided by a health centre that also serves surrounding communities. In Maamobi, primary health care service is provided by the Maamobi General Hospital located in the heart of the Maamobi township and also serves several communities in its vicinity. In each site, two independent, HIV-naïve, groups of pregnant women were enrolled in cross-sectional surveys; one survey targeted pregnant women attending their first ANC to get an overview of the appearance of women at first ANC and their characteristics, while the other targeted pregnant women attending the maternity wards for delivery, and was used to study the IPTp coverage and its impact.

### Ethics statement

All experimental protocols were approved by the Institutional Review Board (IRB) of the Noguchi Memorial Institute for Medical Research (NMIMR) and the Ethics Review Committee of the Ghana Health Service (GHS). Written informed consent was obtained from each participant before they were admitted into the study. All methods were carried out in accordance with the relevant guidelines and regulations.

### Data and biological sample collection

Standardised questionnaires and checklists were used to collect demographic, clinical and ultrasound data for the cohort at the first ANC. As standard practice, pregnant women attending ANC in these two centres are given IPTp-SP under direct observation, and the health staff make entries into their booklets after this directly observed therapy (DOT). Three (3) ml of peripheral blood were collected into EDTA vacutainer tubes before administration of IPTp-SP. Malaria screening by light microscopy and haemoglobin (Hb) level determinations by Hemocue® were performed.

For the cohort at delivery, standardised questionnaires and checklists were also used to collect demographic and clinical data. Information on IPTp-SP uptake was obtained from participants’ ANC booklets. Three (3) ml of peripheral blood and 3 ml of cord blood were collected into separate EDTA vacutainer tubes, and peripheral Hb level measured. Thick and thin blood smears were made for microscopic detection of *Plasmodium*. A piece of delivered placenta from the mother’s side was cut and blots made from it.

All blood samples were centrifuged, and the separated components (plasma and red blood cell pellets) were stored at −80 and −20 °C respectively for further analysis.

### Parasitological determinations by microscopy and real-time PCR assay

Thick and thin blood films were read for *Plasmodium* species according to standard, quality-controlled procedures^27^. DNA extraction was carried out on red blood cell pellets using the QIAamp DNA blood Mini Kit (Qiagen, France) according to the manufacturer’s recommendations. *P. falciparum* parasites were detected and quantified in duplicate by real-time quantitative polymerase chain-reaction (qPCR) targeting the 18S rDNA (18SqPCR)^27,28^ and the acidic terminal segment (ATS) of *P. falciparum var* gene^29^. For 18SqPCR the detection was carried out on 40 amplification cycles. Parasitaemia was further determined by extrapolation of cycle thresholds (Ct) from a standard curve of 3D7 *P. falciparum* infected erythrocyte culture, and generated simultaneously on each plate. For the detection of parasite DNA with ATSqPCR, amplification was extended over 45 cycles as previously described^29^. Purified DNA from 3D7 parasite strain was used as a positive control while a negative control with no DNA template was run in all reactions.

### Antimalarial drug level measurements

Plasma sulfadoxine and pyrimethamine levels were measured by liquid chromatography coupled to tandem mass spectrometry (TSQ Quantum Ultra, Thermo Fisher, France) with electrospray ionisation in positive mode using an Atlantis T3 (100 mm × 2.1 mm, 3 µm) column (Waters, France). Separation was assessed using a 0.30 ml/min flow rate and a gradient mode using two solvent phases, A (water/methanol/formic acid, 95/4.9/0.1% v/v) and B (methanol/formic acid 99.9/0.1% v/v). Specific multiple reaction monitoring (MRM) transitions were selected for detecting sulfadoxine (311.10/245.10) and pyrimethamine (249.10/233.10). Quinide-d3 (328.09/163.04) was used as an internal standard. Separation was achieved in less than 10 min. Using 96-well micro plates (OASE Waters, France), 100 µl of plasma were mixed with 300 µl of acetonitrile containing quinide-d3 (50 ng/ml) as internal standard proteins and phospholipids were eliminated by positive pressure (20 psi during 1 min.). Eluents were evaporated at room temperature, and dry residues were dissolved in 20 mM ammonium formate buffer with formic acid (0.5% v/v) before 10 µl was injected into the system. Method was linear between 10 and 10000 ng/ml for sulfadoxine and between 10 and 1000 ng/ml for pyrimethamine. Lower limit of quantification was 10 ng/ml for both sulfadoxine and pyrimethamine. Upper limits of quantification were 25000 and 5000 ng/ml for sulfadoxine and pyrimethamine, respectively.

For homemade and external controls (WWARN controls), coefficients of variation were below 10%, and bias values were ±10%. Appropriate dilutions were performed in blank serum for all samples and concentrations higher than 10,000 ng/ml for sulfadoxine or higher than 1,000 ng/ml for pyrimethamine and re-analysed.

### Statistical analysis

At delivery, the primary exposure variable of interest was IPTp-SP usage, and it was grouped into 3 categories; women who did not receive any SP, women who received 1 or 2 doses, and women who received ≥3 doses of SP. We analyzed the beneficial effect of SP on birth weight based on the number of SP intakes using a multivariate linear regression model. The risk of *P. falciparum* infection during pregnancy both at first ANC and at delivery was analyzed using a multivariate logistic regression model. To evaluate the association between SP uptake and residual SP level, we performed a logistic regression after categorizing SP into a binary variable based on the median level. In these analyses, the adjustment factors were gravidity (primigravidae or multigravidae), infection status (positive or negative by PCR), gestational age and SP intake information from the ANC booklets. The Cohen Kappa test was used to test for concordance between the 18SqPCR and the ATSqPCR methods used in *P. falciparum* detection. All statistical analyses were done with Stata(version 13.0). All tests were conducted using 0.05 level of significance.
